# Hospital Hygiene

**Published:** 1873-06

**Authors:** J. De Zouche

**Affiliations:** Albany, N. Y.


ART. II.—*Hospital Hygiene.* By J. De Zouche, M. D., of Albany. Read before the Albany County Medical Society, May 14th, 1873.

In the earlier times, before medical colleges had existence or boards of health had a place among existing institutions, there were, nevertheless, some excellent ideas extant on the subject of health—ideas that have not been much improved on, during the last two or three thousand years. Moses, the law-giver of the Hebrews, gave very explicit and excellent directions concerning food, cleanliness and general quarantine regulations, many of which might be followed with advantage even now.

There was something other than mere fanaticism in the Jewish discrimination between clean and unclean beasts, and in their proscription of certain parts of even clean beasts as articles of food. Then their laws in regard to the purifying of parturient women, the cleansing of lepers, the isolation of all infectious or offensive cases, the removing of the dead from contact with the living, the frequent ablutions insisted on in all cases, *all* prove that Moses was a sanitarian of the most enlightened stamp, a worthy leader of the people in their struggle for a higher life.

The name of my subject, "Hygiene," recalls other peoples than Jewish. However irrationally the old Greeks and Romans *lived*, they furnish us with many valuable hints on life, health, and many other subjects.

Their goddess of health, Hygeia, was the daughter of a physician, Esculapius, who was the son of the god Apollo, who was, you may remember, the leader of that celebrated band, "the tuneful nine." To be sure all this is mythological. The physician does not always spring from deity, nor is his daughter invariably a goddess of health. Still there is enough truth in the myth, to warrant its acceptance even now as an article of faith.

A subtle meaning underlies the thought that a *goddess* presides over health. That she sprung from a physician may mean that she was the legitimate offspring of the most advanced expression of sanitary science. But we must leave mythology, though attractive, and come down to facts, however dry.

There is, or should be, nothing special in *hospital* hygiene: for the means by which health may be conserved or promoted should have, and really do have a universal application. The hygienic measures essential in an hospital are demanded in every private dwelling; only perhaps there is more urgent need of them in the former, where so many are congregated under one roof, and all with health more or less impaired.

The site of an hospital should invariably be in the healthiest part of the city or district, that, in fighting disease, the physician should not have native enemies in the camp to contend with. It is of little avail to treat intermittent fever in a low marshy neighborhood—the home of malaria.

"The site of an hospital," wrote the late Edwin M. Stanton, "should be well drained, with a subsoil of gravel. The situation should be elevated as much as possible from marshes or other sources of malaria, and must have a convenient supply of pure water.

In an elevated situation we have the requisite condition for good natural drainage; but even *there* we must have the subsoil of *gravel*, for if it be of *clay* there is sure to be an injurious amount of moisture, and this subsoil moisture plays no mean part in the inception and continuance of many infectious and epidemic diseases.

The high situation and natural drainage insisted on, is not intended to supercede artificial drainage, but rather to divide or supplement the work of keeping the site as free from dampness as possible. Hammond urges strongly the necessity of examining into the character of the soil and subsoil, for if the site be chosen regardless of these points, disease, either fevers, bowel affections, rheumatism, or catarrh will inevitably be produced." Besides the natural drainage which is the result of an elevated situation, we have, as a second important result, a much freer circulation of air, than is possible on a low level, and this is almost as important as the drainage. Stagnant air is as much to be feared as stagnant water.

The drains leading from the building should be so planned that their course towards the main sewer would be as direct as possible, without unnecessary angles or tortuousness. In their construction it would be well to remember that *stagnation is death.* There should always be provision for a stream of water passing through to carry the sewage with it, and prevent any accumulation of organic refuse. There should be, at the same time, good drain ventilation, the ventaduct leading to the top of the building, that the noxious sewer gases may not invade the house, bringing typhoid, and other diseases in their train.

The best plan for hospital buildings, according to the best informed authorities, embraces what is known as the pavilion system —that is, separate little buildings or wards, totally separated from each other, merely accessible by a common corridor. The space between any two wards should be sufficient to prevent the possibility of the spread of contagious diseases or matter through the open windows. With this form and arrangement of an hospital we secure two very important points—a free circulation of air and unobsructed light. It would be all the better if these separate little buildings were only *one story high*, but space is too valuable in situations suitable for an hospital, to permit of so great a sanitary luxury. Where there are two or three stories, the separation of each from all the others should be as complete as possible.

There should not be any openings directly from the lower wards to the upper. The stairways should be so placed that the foul air from below could not ascend that way to mingle with the air of the upper wards. The true principle of ventilation must provide for all impure air being conveyed away out of the building from each floor. When the windows of the lower wards are open I would recommend that over each, there should be a projecting canopy arranged in such a way as to lead the impure air from the ward as far out from the face of the building as possible, so that no portion of it might enter the open windows of the upper wards. The floors should be far more solid and air-tight than contractors usually make them, and between the rafters there should be a packing sufficient to bar the passage of air from below at the same time that it would deaden the sounds of feet or other noises above.

In an address to the Southampton Medical Society, Dr. Langstatt lays great stress on the importance of having a great number of small separate rooms, instead of the few large ones which we constantly find in large hospitals; but as we cannot hope to see any great change in the construction of existing institutions of the kind, he proposes to make the best of what we have. He, has found, as well as other physicians whom he mentions, that the floors and walls of an hospital after continued use absorb matter which leads to the outbreak and spread of disease, and that it behoves to apply to them a non-absorbent material, and one that can readily be cleaned. Recognizing the great value of lime as a purifier, he nevertheless believes that white-washed walls very soon absorb the poison from the sick-bed, and require to be frequently renewed. The best method, he believes, of rendering hospital walls and ceilings non-absorbent, is to paint them, on a smooth surface with several coats of paint, and finally varnish them.

The floors might be made non-absorbent by painting them with hard paraffine, previously melted in an iron vessel, and ironed in, with a box iron heated from the interior with charcoal.

Under no circumstances should there be paper on the walls of an hospital ward, nor carpet on the floor, as they absorb and retain for an indefinite period the poisonous germs that arise from disease, and render the room unsafe for future occupants. Neither should patients, nurses or house physicians wear woolen articles of clothing, as they tenaciously hold the "hospital smell," which is at least suggestive of contagion.

In ordinary dwellings where the inmates are, as a rule, healthy, the ordinary means of ventilating, by windows and doors, answers the purpose very well; but in an hospital where medical and surgical diseases prevail, and the air becomes laden with the foul exhalations from every variety of decaying tissue, it is of the utmost importance to take advantage of every means by which pure air may enter and the impure escape most effectively. Now pure air, as a rule, enters a living room at the lowest openings, whilst the air which has become vitiated by having passed through the lungs, or contaminated by contact with the exhalations from the surface of the diseased body, ascends to the top of the room, and will escape, *more or less completely*, by the highest openings. I qualify the statement because I believe it will not *wholly* escape unless the highest openings are at the highest point in the room—on a level with the ceiling. The lower openings should be at or near the level of the floor. The exhaled air is largely composed of carbonic acid gas which, at the same temperature ot the pure air, is much heavier than it—volume for volume. When first exhaled, being expanded by heat, its specific gravity is less and it ascends, to escape measurably by such upper openings as there may be; but if proper provision has not been made above for its departure, at this time, it quickly condenses and sinks to the lower part of the room, where it is destined to struggle with the pure air, provided there are inlets.

Now if there are openings at the level of the floor, on all four sides of the room, there will be little opportunity for the accumulation of this poisonous gas, which would escape on that side of the room which sailors call "leeward" impelled by the pure air entering at windward." The upper openings in the room should be somewhat larger than the lower, for obvious reasons, and I think it important that they should be at the *level of the ceiling*. Where this is neglected there will always be a reserve of deleterious gases found hovering about the angles, and tainting the whole upper stratum of air in the room, to the great detriment of the patient to whom the oxygen of the pure air is even more essential than calomel or quinine.

Water closets should invariably be placed at the *boundary walls,*

each with a window admitting abundance of sunlight and giving the opportunity for the ingress of pure air and the egress of noxious air; and all this besides the ventilation proper to the soil pipe. Water-closets should never be constructed in dark corners in the centre of a building, for however thorough the system of ventilation may be, there will always be at least a strong suspicion of impurity in the air—a suspicion that will hardly bear the test of chemical analysis.

A bath-room on each floor to which patients should be introduced on admission and obliged to use at least twice a week during their stay in the hospital, will prove a valuable auxiliary in the sanitary work. With good drainage, a constant supply of pure air, and a persistent regard for personal cleanliness, the doctor's work would be simplified, and the proportion of "good recoveries" be greatly increased.

The regulation of the temperature is a matter of very great importance, and, I may add, of great difficulty too, with such heating apparatus as we find generally in use. So long as the air we breathe is kept below 65° Fahrenheit it acts as a stimulant to the lungs, which accordingly work with more vigor to keep up the heat of the body in the natural way. Combustion goes on more or less perfectly, and the effete products of decay are rapidly expelled from the system. But when we permit the temperature of the living room to reach 70° or upwards, nature recognizes the wrong and protests against it. The lungs, to be sure, do not *cease* to act, but they seem to lose tone and work more languidly, the combustion of effete material going on so slowly, much of it lingers in the system to poison the blood and deaden the fire of life. The effect of continued high temperature, especially when artificial, on even healthy people, is a relaxation of muscular fibre, superinducing a listlessness almost amounting to paralysis of the will.

Special care should be taken to classify patients according to their diseases and the measure of their sickness. Cases of erysipelas, hospital gangrene, some fevers and contagious diseases which may have found their way into the hospital, or become developed there, should be isolated in the upper story of the hospital where it consists of *more than one story.*

During the prevalence of erysipelas, hospital gangrene, syphilis, etc., sponges should never be used; tow or rags, which should be burned after once using, might be substituted with advantage in dressing wounds and ulcers.

In regard to the size of hospitals there is a growing belief that the larger they are the worse it is for the patient. Amputations become more dangerous and fatal in proportion as the hospitals in which they are performed increase in size. By separation and isolation the patients recover with more certainty. The safety is in segregation, the danger in aggregation. In the Edinburg Monthly Journal of Medical Science for November, 1838, Sir James Y. Simpson wrote on this subject an article from which I will quote one or two passages.

He says: "Perhaps one of the most weighty and momentous questions to which the physician, the surgeon and the accoucheur can direct his attention is the proper reconstruction and arrangement of our hospitals. The vast importance of the subject depends upon *this* point—that it involves the study and rectification of influences that set utterly at defiance all the proudest advances of practical medicine."

Sir James goes on to speak of the signal and striking progress made by surgery during the last 50 or 100 years in various ways and in various directions, and yet, notwithstanding all that, the proportion of *fatal results of operations* has been steadily increasing. "Amputation, for instance, as an operation, has, like many other operations, been mightily improved, in the modes of its performance, in the modes of arresting attendant hemorrhage, in the modes of dressing the stumps, etc.; but still in these hospitals (the infirmaries of Edinburg and Glasgow) the mortality from limb-amputations has, since the last century, become increased instead of diminished. The increase is traceable, I believe, chiefly or entirely to our system of huge and colossal hospital edifices, and to the hygienic evils which that system has hitherto been made to involve. * * * I have often stated and taught that if our present medical, surgical and obstetrical hospitals were changed from being crowded palaces, with a *layer* of sick on each *flat*, into villages or cottages, with one or at most two patients in each room, a great saving of human life would be effected."

To substantiate what he says, Sir James gives facts and figures which throw much light on the subject. The following are suggestive:

Table showing the proportionate *mortality of limb amputations* in Great Britain, as regulated by the size of the hospital and the degree of aggregation or isolation in which the patients are placed:

| *Size of Hospitals, etc.* | *Death-rate.* |
|---|---|
| (1) In hospitals containing 300 to 600 beds . | 1 in 2½, die. |
| (2) In hospitals containing 100 to 300 beds . . . | 1 in 4, die. |
| (3) In hospitals containing 25 to 100 beds . . | 1 in 5½, die. |
| (4) In cottage hospitals containing under 25 beds . . | 1 in 7, die. |
| (5) In isolated rooms in country practice . . | 1 in 9, die. |

"These few figures teach a lesson of vast import in relation to hospital hygiene, and yet they seem to discourse so plainly as to require no comment."

In studying the sickness and death-rate of communities, and all the conditions under which the statistics relating to them grow we furnish ourselves with the weapons necessary to fight and conquer a thousand ills that decimate the population of our cities. The proportion is large of those who die from preventable causes, to some of which I have referred in this paper.

I have thus written down, without much system, such suggestions as occurred to me on this very important subject, which I think as worthy of our best consideration, as any that the physician and surgeon have to master.

